# Which Factors, Smoking, Drinking Alcohol, Betel Quid Chewing, or Underlying Diseases, Are More Likely to Influence the Severity of COVID-19?

**DOI:** 10.3389/fphys.2020.623498

**Published:** 2021-01-18

**Authors:** Rui Zhong, Lingxia Chen, Qiong Zhang, Binbin Li, Yanfang Qiu, Wei Wang, Dongyi Tan, Yanhui Zou

**Affiliations:** ^1^Hunan Cancer Hospital/The Affiliated Cancer Hospital of Xiangya School of Medicine, Central South University, Changsha, China; ^2^The First People’s Hospital of Yueyang, Yueyang, China; ^3^Key Laboratory of Molecular Radiation Oncology Hunan Province, Xiangya Hospital, Central South University, Changsha, China

**Keywords:** SARS-cov-2, smoking, alcohol, betel quid chewing, underlying diseases, route of transmission, diabetes mellitus, COVID-19

## Abstract

The global outbreak of the coronavirus disease 2019 (COVID-19) pandemic occurred in late 2019 and early 2020. The factors that influence disease severity should be of clinical concern. Existing findings on the effects of smoking on COVID-19 are also controversial and need to be confirmed by further research. In addition, the effects of alcohol consumption and betel quid (BQ) chewing on COVID-19 are unclear. The aim of this study was to examine the demographic characteristics of COVID-19 patients and the effects of smoking, drinking, BQ chewing, and underlying diseases on the severity of COVID-19. A retrospective study was conducted on 91 patients with confirmed cases of COVID-19 hospitalized in Yueyang, Hunan Province, China from 21 January to 8 March, 2020. Patient demographic data, and information on smoking, drinking and BQ chewing, and underlying diseases were extracted from the patient electronic medical records (EMR) and telephone interviews. The chi-square test was used to conduct a univariate analysis of the factors influencing the severity of COVID-19, and ordinal logistic regression analysis was used to identify the factors related to the severity of COVID-19. The results showed that the rates of smoking, drinking and BQ chewing were 15.4, 26.4, and 7.1%, respectively, there was no significant relationship between these lifestyle factors and the severity of COVID-19 (*P* > 0.05). However, underlying diseases such as diabetes [odds ratio (OR) = 7.740, 95% confidence interval (CI):1.000–60.740, *P* = 0.050], source of infection (OR = 0.180, 95% CI: 0.030–0.980, *P* = 0.049), and employment status (retired/unemployed vs. employed: OR = 29.430, 95% CI, 1.050 – 822.330, *P* = 0.047) were significant independent predictors of severe COVID-19 infection. These individuals should be informed of methods to increase personal protection, and doctors should prevent these individuals from developing serious diseases. It is important to pay attention to the source of infection and timely medical treatment. This study showed that the clinical classification of COVID-19 was associated with patients with diabetes, source of infection, and retired/unemployed. Therefore in the clinical practice of COVID-19 should be more concern these factors. Although no statistical significance was found in smoking, drinking alcohol, BQ chewing, and severity of COVID-19 patients, more studies have confirmed that are harmful and risk factors for underlying diseases in the population. Health authorities should formulate policies to publicize the harmful effects of smoking, drinking, and betel nut chewing and promote a healthy lifestyle.

## Introduction

Since the severe acute respiratory syndrome coronavirus 2 (SARS-CoV-2) outbreak began in December 2019, it has rapidly swept around the world. The resultant disease, coronavirus disease 2019 (COVID-19), was officially declared a pandemic by the World Health Organization on 11 March 2020 (Huang C. L. et al., 2020; Tian et al., 2020). Whether the source of infection of COVID-19 is related to the severity of the disease is rarely reported. The source of infection is mainly patients displaying symptoms of COVID-19, although asymptomatic infected persons and patients in the incubation period are also contagious to some extent (Special Expert Group for Control of the Epidemic of Novel Coronavirus Pneumonia of the Chinese Preventive Medicine Association, 2020). SARS-CoV-2 is mainly transmitted through droplets and close contact and is also spread by aerosols (Morawska and Milton, 2020; National Health Commission and National Administration of Traditional Chinese Medicine, 2020; Setti et al., 2020). Aerosol transmission needs to be in a relatively closed space. When the aerosol containing the virus reaches a certain concentration, it is likely to cause infection if inhaled by healthy people.

Factors contributing to the severity of the disease have attracted attention. Age, underlying diseases, employment are considered important risk factors that are relevant to disease severity in COVID-19 patients (Chen N. et al., 2020; Huang C. L. et al., 2020; Nandy et al., 2020; Wang D. et al., 2020; Wang X. et al., 2020). Chen R. C. et al. (2020) studied 1,590 COVID-19 patients in China and found that in patients aged 65 years or older, coronary heart disease, cerebrovascular disease, dyspnea and other independent risk factors were related to fatal outcomes. Guan et al. (2020a) reported that compared with patients without underlying diseases, COVID-19 patients with more types of underlying diseases had a worse prognosis. Several Studies have reported that, 20–51% of COVID-19 patients have at least one underlying disease, among which diabetes (10–20%) is the most common, followed by hypertension (10–15%) and other cardiovascular and cerebrovascular diseases (7–40%) (Chen N. et al., 2020; Huang C. L. et al., 2020; Liu K. et al., 2020). Aggarwal et al. (2020) conducted a meta-analysis of 12 studies, involving a total of 2,564 patients, of whom 265 (10.3%) had a history of diabetes exacerbating the clinical progression of COVID-19. Yu et al. (2020) conducted a retrospective study of 1,463 COVID-19 patients and found that age, male sex, and a history of diabetes were independent risk factors for COVID-19 mortality. Liu J. et al. (2020) conducted a retrospective analysis of 48,814 confirmed cases of COVID-19 in the Hubei Province, and found that employment status (retired/working at home) is also an independent risk factor for the severity of COVID-19. However, these findings need to be confirmed by further cohort or prospective studies.

Mao et al. (2020) analyzed the epidemiology of 67 COVID-19 clusters (including 226 confirmed patients) in Sichuan Province, China, and found that families (68.66%) and living with family (60.87%) were the main exposure. However, whether the cause of exposure is related to the severity of the disease is rarely reported.

The relationship between smoking and COVID 19 is controversial. Many studies have shown that the smoking rate of COVID-19 patients is lower than that of the overall smoking rate of the population (Guan et al., 2020b; Zhang J. J. et al., 2020). According to the results of the 2018 Global Adult Tobacco Survey, the smoking rate of Chinese adults is 26.6%, and the average daily smoking amount is 16.0 cigarettes (Global Adult Tobacco Survey [GATS], 2020). Systematic reviews (Farsalinos et al., 2020a; Patanavanich and Glantz, 2020) have shown that the current smoking rate of 5,960 COVID-19 inpatients in China was 1.4–14.6%.

Angiotensin-converting enzyme 2 (ACE 2) is the main receptor of SARS-CoV-2 entering human cells. Studies have shown that smoking can cause high expression of ACE2, which may lead to poor prognosis of COVID-19 (Brake et al., 2020; Leung et al., 2020). Systematic reviews by Vardavas and Nikitara (2020) suggest that smoking is most likely associated with rapid progression and adverse outcomes of COVID-19. Patanavanich and Glantz (2020) showed that smokers were 1.91 times more likely to develop severe COVID-19 than non-smokers.

However, some scholars have proposed that ACE 2 upregulation does not necessarily equate to increased susceptibility or disease severity, and nicotine in tobacco may even have a therapeutic effect (Farsalinos et al., 2020a,b). Guan et al. (2020b) studied 1,099 hospitalized COVID-19 patients in China and found that 16.9, 5.2, and 77.9 the patients with severe COVID-19were current smokers, former smokers, and never smoked respectively. Univariate analysis was statistically significant (*P* < 0.001). Simons et al. (2020) included a total of 233 studies in the systematic review and found that current smokers appeared to have a reduced risk of SARS-CoV-2 infection compared with those who had never smoked, whereas former smokers appeared to have an increased risk of hospitalization for COVID-19, increased disease severity, and death, although these associations were causally unconfirmed. Although the relationship between smoking and COVID-19 severity is uncertain, there is growing evidence that smokers have a higher risk of serious illness and death with COVID-19 (World Health Organization [WHO], 2020). Although the prevalence of smoking among patients is low, the relationship between smoking and COVID-19 is still worth exploring, and several studies have confirmed that smoking exacerbates COVID-19.

BQ chewing, a centuries-old practice in China, especially in the southern province of Hunan, is as much a stress reliever, social activity or hobby for locals as smoking and drinking. The latest literature reports that there are approximately 0.6–1.2 billion people chewing areca worldwide, with no limitations for children (Gupta and Warnakulasuriya, 2002). Studies have shown (Garg et al., 2014; Mehrtash et al., 2017) that BQ affects almost all organs of the human body, including the brain, heart, lung, gastrointestinal tract and reproductive organs. It also affects the immune system, such as inhibiting T cell activity and reducing cytokine release.

Too much alcohol consumption can also be harmful. The World Health Organization (World Health Organization Regional Office for Europe, 2020) stresses that every drink, whether beer, liquor, or other alcoholic beverage, is harmful. Heavy drinking, in particular, weakens the body’s immune system, making it less able to cope with infectious diseases. In Hunan, China, more than 10% of people over 15 chew BQ regularly, and most also smoke and drink alcohol. The latest research shows that the smoking rate of adults aged 15 and older in the Hunan Province is 28.3%, and that of men is 54.4% (Qi et al., 2020). Currently, there are few studies on the effects of alcohol consumption and BQ chewing on COVID-19, which is also a focus of attention. Therefore, this study analyzed the factors influencing the severity of COVID-19 among patients hospitalized in a hospital in Hunan, China, to provide a reference for the prevention and treatment of COVID-19.

## Materials and Methods

### Study Setting and Participants

The study was conducted in a tertiary hospital in Hunan Province, Yueyang City, which is qualified to receive and treat COVID-19 patients.

All participants with COVID-19 received treatment at The First People’s Hospital of Yueyang from January 21 to March 8, 2020. Inclusion criteria: COVID-19 was diagnosed. Exclusion criteria: Deaths due to non-COVID-19.

### Participants’ Material Collection

The researchers used medical records and telephone interviews to collect the data. The survey mainly included demographic data of the subjects, clinical classification of COVID-19, smoking, drinking, and BQ chewing behaviors. Patient data were extracted from the EMR system of the hospital by professionally trained researchers. If the data were not entered, the researchers would conduct a phone interview to supplement the accurate information. Any problems found were corrected in a timely manner, and supplementary investigations were conducted on non-conforming items. The input data were doubled, carefully checked, and then analyzed.

### Variable Definitions and Criteria

(1) Disease severity classification criteria for COVID-19: Clinical typing. According to the Diagnosis and Treatment Guidelines by the National Health Commission, the patients were divided into mild type, common type, severe type, and critical type.

Mild Type: Clinical symptoms were mild, and no pneumonia was found on imaging.

Common type: With fever, respiratory symptoms, etc., and imaging findings of pneumonia.

Severe type: Adults meet any of the following criteria: A. Onset of shortness of breath, RR ≥ 30 times/min; B. In the resting state, oxygen saturation ≤93% when inhaling air; C. Partial arterial oxygen pressure (Pa02)/oxygen absorption concentration [(Fi02) ≤ 300 mmHg, 1 mmHg = 0.133 kPa].

Critical type: Participants NA.

(2) Smoker is defined as those who smoke ≥1 cigarette/d for more than half a year. Questions included whether smoking, starting age, and the average number of cigarettes consumed per day.

(3) Drinking alcohol was defined as drinking white wine ≥once per week for more than 1 year (The amount of alcohol consumed in alcohol is 52 degrees of high-alcohol liquor, and 75 ml of low-alcohol liquor is equivalent to 50 ml of white wine; 750 ml beer is equivalent to 75 ml white wine; 200 ml wine or yellow rice wine equals 50 ml white wine). Questions included whether drinking alcohol, starting age, what wine consumed and the average amount of alcohol consumed per day.

(4) BQ chewing is defined as BQ chewing ≥once a day for more than 3 months. Questions included whether chewing betel quid, starting age, and the average number of BQ chewing per day.

(5) Underlying diseases: According to the diagnosis and confirmation of the patient’s medical records, information on six common underlying diseases was collected: hypertension, diabetes mellitus, coronary heart disease, cerebral infarction, hyperlipidemia, and renal insufficiency.

### Statistical Analysis

Data were entered and analyzed using SPSS version 18.0 (SPSS, Chicago, IL, United States). Descriptive statistics were calculated for general information, smoking, drinking alcohol, BQ chewing. We performed univariate analyses with chi-square tests between the clinical typing of COVID-19. Ordered logistic regression analysis was used to identify the factors related to clinical typing of COVID-19. Odds ratios (OR’s) and 95% confidence intervals (CI’s) were evaluated in multivariable analysis. A *P*-value ≤ 0.05 denoted statistical significance.

## Results

### Demographic Information and Characteristics of the Cases

**FIGURE 1:**
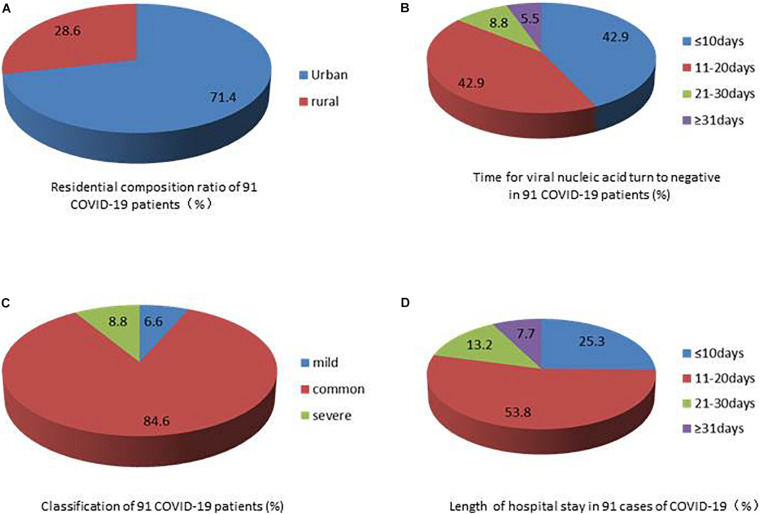
**(A)** Residential composition ration of 91 COVID-19 patients (%). **(B)** Time for viral nucleic acid turn to negative in 91 COVID-19 patients (%). **(C)** Classification of 91 COVID-19 patients (%).**(D)** Length of hospital stay in 91 cases of COVID-19 (%).

A total of 91 COVID-19 patients who met the diagnostic criteria were enrolled in this study. There were 46 men (50.5%) and 45 women (49.5%), the minimum age was 3 months, the maximum age was 76 years, and the mean age was 47.3 ± 16.7 years. 72 (79.1%) were married, 19 (20.9%) were single. The employment results showed that the service category, 24 (26.3%) and the retired/unemployed, 22 (24.2%) were in the majority. The distribution of education level was primary school and below, 36 (39.6%), junior high school, 31 (34.1%), senior high school, 18 (19.8%), and junior college and above, 6 (6.5%). Urban residents accounted for 65 (71.4%), while rural residents accounted for 26 (28.6%) (Figure 1A). The maximum number of days of virus negative conversion was 42 days, and the minimum was 4 days. The average was 13.8 ± 7.8 days (Figure 1B). The patients had a history of living or traveling in Hubei or had contact with COVID-19 patients in Hubei 38 (41.8%) and with COVID-19 infection due to a local or family gathering 53 (58.2%). The classification of 91 COVID-19 cases were 6 (6.6%) mild, 77 (84.6%) common, and 8 (8.8%) severe, respectively (Figure 1C). The longest hospital stay was 43 days, and the shortest was 5 days. The average length of hospital stay was 16.1 ± 7.6 days (Figure 1D and Table 1).

**TABLE 1:** Relationship between patient characteristics and disease severity of COVID-19.

Variables	Total *n* = 91(%)	Mild *n* = 6(%)	Common *n* = 77(%)	Severe *n* = 8(%)	P*
Gender					**0.155**
Male	46 (50.5)	5 (10.9)	36 (78.2)	5 (10.9)	
Female	45 (49.5)	1 (2.2)	41 (91.1)	3 (6.7)	
Age (years)	**47.3 ± 16.7**				**0.001**
≤30	16 (17.6)	5 (31.2)	11 (68.8)	0 (0.0)	
31–64	60 (65.9)	1 (1.7)	53 (88.3)	6 (10.0)	
≥65	15 (16.5)	0 (0.0)	13 (86.7)	2 (13.3)	
Habitation					**0.432**
City	65 (71.4)	3 (4.6)	57 (87.7)	5 (7.7)	
Rural	26 (28.6)	3 (11.5)	20 (77.0)	3 (11.5)	
Marriage					**0.001**
Single	19 (20.9)	5 (26.3)	14 (73.7)	0 (0.0)	
Married	72 (79.1)	1 (1.4)	63 (87.5)	8 (11.1)	
Education					**0.019**
Primary school or less	36 (39.6)	5 (13.9)	24 (66.7)	7 (19.4)	
Middle school	31 (34.1)	0 (0.0)	31 (100.0)	0 (0.0)	
High school	18 (19.8)	1 (5.6)	16 (88.8)	1 (5.6)	
College school or above	6 (6.5)	0 (0.0)	6 (100.0)	0 (0.0)	
Occupation					**0.000**
Retired/unemployed	22 (24.2)	0 (0.0)	18 (81.8)	4 (18.2)	
Service personnel	24 (26.3)	0 (0.0)	22 (91.7)	2 (8.3)	
Other workers	38 (41.8)	2 (5.3)	34 (89.4)	2 (5.3)	
Student	7 (7.7)	4 (57.1)	3 (42.9)	0 (0.0)	
Underlying disease					**0.017**
Yes	26 (28.6)	1 (3.8)	19 (73.1)	6 (23.1)	
No	65 (71.4)	5 (7.7)	58 (89.2)	2 (3.1)	
Infection source					**0.019**
Hubei related infection	38 (41.8)	0 (0.0)	32 (84.2)	6 (15.8)	
Local infection	53 (58.2)	6 (11.3)	45 (84.9)	2 (3.8)	
Length of stay	**16.1 ± 7.6**				**0.231**
≤10	23 (25.3)	1 (4.3)	22 (95.7)	0 (0.0)	
11–20	49 (53.8)	3 (6.1)	39 (79.6)	7 (14.3)	
21–30	12 (13.2)	2 (16.7)	10 (83.3)	0 (0.0)	
≥31	7 (7.7)	0 (0.0)	6 (85.7)	1 (14.3)	
Virus negative days	**13.8 ± 7.8**				**0.087**
≤10	39 (42.9)	1 (2.6)	37 (94.8)	1 (2.6)	
11–20	39 (42.9)	3 (7.7)	30 (76.9)	6 (15.4)	
21–30	8 (8.8)	2 (25.0)	6 (75.0)	0 (0.0)	
≥31	5 (5.4)	0 (0.0)	4 (80.0)	1 (20.0)	
Smoking					**1.000**
Yes	17 (15.4)	1 (5.9)	14 (82.3)	2 (11.8)	
No	74 (84.6)	5 (6.8)	63 (85.1)	6 (8.1)	
Smoking daily (cigarette)	**15.7 ± 11.4**				**0.207**
≤10	10 (58.8)	0 (0.0)	8 (80.0)	2 (20.0)	
11–20	4 (23.5)	0 (0.0)	4 (100.0)	0 (0.0)	
21–30	1 (5.9)	0 (0.0)	1 (100.0)	0 (0.0)	
≥31	2 (11.8)	1 (50.0)	1 (50.0)	0 (0.0)	
Smoking duration (years)	**22.9 ± 14.6**				**0.491**
≤10	6 (35.3)	0 (0.0)	6 (100.0)	0 (0.0)	
11–20	1 (5.9)	0 (0.0)	1 (100.0)	0 (0.0)	
21–30	6 (35.3)	0 (0.0)	5 (83.3)	1 (16.7)	
≥31	4 (23.5)	1 (25.0)	2 (50.0)	1 (25.0)	
Alcohol					**0.547**
Yes	24 (26.4)	1 (4.2)	22 (91.6)	1 (4.2)	
No	67 (73.6)	5 (7.5)	55 (82.1)	7 (10.4)	
Amount of drinking (ml)	**147.9 ± 75.9**				**0.516**
≤100	10 (41.7)	0 (0.0)	10 (100.0)	0 (0.0)	
101–200	9 (37.5)	1 (11.1)	7 (77.8)	1 (11.1)	
≥201	5 (20.8)	0 (0.0)	5 (100.0)	0 (0.0)	
Drinking duration (years)	**19.4 ± 13.5**				**0.261**
≤10	10 (41.7)	0 (0.0)	9 (90.0)	1 (10.0)	
11–20	7 (29.2)	0 (0.0)	7 (100.0)	0 (0.0)	
21–30	3 (12.5)	1 (33.3)	2 (66.7)	0 (0.0)	
≥31	4 (16.6)	0 (0.0)	4 (100.0)	0 (0.0)	
Chewing betel nut (nut)	**8.7 ± 5.9**				**0.765**
Yes	6 (7.1)	0 (0.0)	6 (100.0)	0 (0.0)	
No	85 (92.9)	6 (7.1)	71 (83.5)	8 (9.4)	
Chewing betel nut duration (years)	**5.5 ± 4.0**				

*Monte Carlo *P* < 0.05.

### The Subjects’ Behaviors of Smoking, Alcohol, and BQ Chewing

Of the 91 COVID-19 patients, 17 were smokers (15.4%); Age at which to start smoking the youngest and the oldest were 14 and 46 years old, respectively. The smoking duration was 22.9 ± 14.6 years, and a smoking duration of more than 10 years accounted for 64.7%. The average daily smoking amount was 15.7 ± 11.4 cigarettes, and 41.2% of those had a daily smoking amount of greater than 10 cigarettes. Twenty-four patients were drinkers (26.4%), 4 in 24 drinkers were often drinkers, and the rest were social drinkers. The average duration of drinking was 19.4 ± 13.5 years, and for 58.3%, the duration was longer than 10 years. Difference between urban and rural drinking duration (year), Monte Carlo *P* = 0.028, urban COVID-19 drinkers (80%) had been drinking for more than 10 years. Rural COVID-19 drinkers (77.8%) had been drinking for less than 10 years. The average consumption of alcohol was 147.9 ± 75.9 ml per time, and approximately 58.3% of those consumed more than 100 ml per time. Of the 91 COVID-19 patients, 6 patients were BQ chewers (7.1%), all in the common type group. The average duration of BQ consumption was 5.5 ± 4.0 years, and the average daily consumption of BQ was 8.7 ± 5.9. The results showed that there were no statistically significant differences in smoking, drinking, and BQ chewing characteristics with COVID-19 clinical typing (*P* > 0.05).

### Relationship Between Clinical Typing of COVID-19 and Demographic Factors

Among the 91 COVID-19 patients, the clinical classifications were 6.6, 84.6, and 8.8% for mild, common, and severe types, respectively. There were no critically ill patients, and all patients recovered and were discharged in good condition. The chi-square test for univariate analysis showed that there were significant differences between age, marital status, education level, occupation, presence or absence of underlying diseases and source of infection and patients’ clinical typing of COVID-19 (*P* ≤ 0.05). The most severe cases of COVID-19 occurred in people aged 65 or older, married people, those with primary school education or below, and those with a history of associated Hubei Province. Severe type COVID-19 was more common in patients with underlying diseases.

### The Relationship Between the Underlying Disease and the Clinical Classification of COVID-19

Of the 91 COVID-19 patients, 26 (28.6%) had underlying disease, of which hypertension 12 (13.2%) was the most common, followed by diabetes 8 (8.8%). The patients with hypertension, coronary heart disease, hyperlipidemia, diabetes, cerebral infarction and renal insufficiency accounted for 13.2, 3.3, 5.5, 8.8, 6.6, and 6.6%, respectively. A total of 12.1% of patients had 2 or more underlying diseases. The results showed that there were significant differences between the clinical types of COVID-19 associated with underlying disease (*P* < 0.05) (Table 2).

**TABLE 2:** Relationship between patient underlying disease and disease severity of COVID-19.

Underlying disease	Total *n* = 91(%)	Mild *n* = 6(%)	Common *n* = 77(%)	Severe *n* = 8(%)	P*
Hypertension					**0.658**
No	79 (86.8)	5 (6.3)	68 (86.1)	6 (7.6)	
Yes	12 (13.2)	1 (8.3)	9 (16.7)	2 (16.7)	
Diabetes					**0.029**
No	83 (91.2)	6 (7.2)	72 (86.7)	5 (6.0)	
Yes	8 (8.8)	0 (0.0)	5 (62.5)	3 (37.5)	
Cerebral infarction					**0.135**
No	85 (93.4)	6 (7.1)	73 (85.9)	6 (7.1)	
Yes	6 (6.6)	0 (0.0)	4 (66.7)	2 (33.3)	
Coronary heart disease (CHD)					**1.000**
No	88 (96.7)	6 (6.8)	74 (84.1)	8 (9.1)	
Yes	3 (3.3)	0 (0.0)	3 (100.0)	0 (0.0)	
Hyperlipidemia					
No	86 (94.5)	6 (7.0)	73 (84.9)	7 (8.1)	0.573
Yes	5 (5.5)	0 (0.0)	4 (80.0)	1 (20.0)	
Renal insufficiency					0.135
No	85 (93.4)	6 (7.1)	73 (85.9)	6 (7.1)	
Yes	6 (6.6)	0 (0.0)	4 (66.7)	2 (33.3)	
Number of underlying diseases					**0.015**
No	65 (71.4)	5 (7.7)	58 (89.2)	2 (3.1)	
1	15 (16.5)	1 (6.7)	12 (80.0)	2 (13.3)	
≥2	11 (12.1)	0 (0.0)	7 (63.6)	4 (36.4)	

*Monte Carlo *P* < 0.05.

### Prediction of Influencing Factors for Clinical Typing of COVID-19 Patients

The influencing factors of clinical typing of COVID-19 patients are discussed in depth. The dependent variables were determined as clinical typing of COVID-19 patients (1 = severe type, 2 = common type, and 3 = mild type), the independent variables were locked as statistically significant factors in univariate analysis, and an ordinal logistic regression model was carried out (α_*in*_ = 0.05, α_*out*_ = 0.10). After adjusting for occupation and marriage in the ordinal regression model, it was found that patients with underlying diseases such as diabetes (OR = 7.740, 95% CI: 1.000–60.740, *P* = 0.050), source of infection (OR = 0.180, 95% CI: 0.030–0.980, *P* = 0.048), and retired/unemployed (OR = 29.430, 95% CI: 1.050–822.330, *P* = 0.047) had more severe COVID-19 infection (Table 3).

**TABLE 3:** Potential multi-factor ordered regression analysis for predicting disease severity of COVID-19.

Variable	b	S_*b*_	Waldχ^2^	*P*	Estimated odds ratio	OR 95% CI
(Covid-19 severity = severe)	−7.591	2.932	6.701	0.010		
(Covid-19 severity = common)	0.247	2.645	0.009	0.926		
Marriage	1.996	1.336	2.230	0.135	7.360	0.540–100.900
Education	0.377	0.377	1.000	0.317	1.460	0.700–3.050
Age	0.054	0.806	0.005	0.946	1.060	0.220–5.120
Comorbidity diabetes mellitus	2.047	1.051	3.794	0.050	7.740	1.000–60.740
Source of infection	−1.733	0.875	3.925	0.048	0.180	0.030–0.980
Occupation (retired/unemployed)	3.382	1.699	3.961	0.047	29.430	1.050–822.330
Occupation (service personnel)	2.980	1.560	3.649	0.056	19.690	0.930–418.970
Occupation (other worker)	1.648	1.342	1.508	0.219	5.200	0.370–72.120
Occupation (student)	0^*a*^					

*Marital status (married/single). Source of infection (local infection/Hubei related infection). ^*a*^Referent.*

## Discussion

### Influence of Factors on the Severity of COVID-19

In this study, age, marriage, education, employment status, underlying diseases and source of infection were significantly different in patients with different COVID-19 severities. However, the final multifactor ordered regression results showed that the underlying diseases of diabetes, source of infection, and employment status (retired/unemployed) were independent influencing factors.

Age was a non-independent influencing factor of multifactor regression analysis, which is inconsistent with the results of other studies (Chen R. C. et al., 2020), we still found out retired/unemployed group was affected with more severe COVID-19 who usually were elder. This may be because the incidence of underlying diseases increases with age, which increases the risk of severe COVID-19 in the elderly. The study also indicated that SARS-CoV-2 infection in infants and young adults is associated with mild and common type illness and these patients generally do not have underlying diseases. The protection babies received may be due to the mother’s antibodies and the antiviral proteins in milk, such as lactoferrin, which are known to prevent coronavirus infection (Root-Bernstein, 2020). Few other studies have reported relationships between COVID-19 and marriage, or education which require further study.

There were 46 men (50.5%) and 45 women (49.5%) in this study, with men being slightly more common than females. This is consistent with the results of Abate et al. (2020) systematic reviews have also indicated that COVID-19 is more common in men than in women.

### Diabetes Is Associated With the Severity of COVID-19

Of the six underlying diseases, we examined (diabetes, hypertension, coronary heart disease, cerebral infarction, renal insufficiency, and hyperlipidemia) diabetes was the only independent factor affecting the severity of COVID-19, which is consistent with the findings of Guo et al. (2020). This may be because people with diabetes have increased serum inflammatory-related biomarkers, such as IL-6, c-reactive protein, and serum ferritin, blood coagulation indexes, and D-dimer levels, Therefore people with diabetes have a potential proinflammatory environment, which can lead rapid deterioration of COVID-19. Other studies have shown that patients with diabetes have a higher risk of respiratory infection due to an impaired immune system, especially reduced innate immunity (Ma and Holt, 2020; Pal and Bhansali, 2020). Even transient hyperglycemia may temporarily affect the innate immune response to infection (Jafar et al., 2016). Huang I. et al. (2020) conducted a meta-analysis of 30 studies including 6,452 patients, and found that diabetes was associated with severity, disease progression, mortality, and ARDS in COVID-19 patients. In addition, ACE2 is highly expressed in pancreatic islets; therefore, the virus may cause sharp fluctuations in the blood glucose levels of patients by destroying the islets, thus affecting prognosis (Yang et al., 2010). Previous studies have shown a strong correlation between higher blood glucose levels and the risk of death from COVID-19. Hyperglycemia is an important independent predictor of mortality, and controlling hyperglycemia during the entirety of hospitalization may reduce the risk of serious illness or death (Coppelli et al., 2020; Lampasona et al., 2020). Zhu et al. (2020) reported in their study that the mortality rate of COVID-19 patients with good blood glucose control (blood glucose fluctuation within 3.9–10.0 mmol/L) was significantly lower than that of patients with poor blood glucose control. Sardu et al. (2020) found that patients with hyperglycemia and COVID-19 who received insulin injection had a lower risk of developing severe disease than those who did not receive insulin injection. Therefore, COVID-19 patients with diabetes should receive careful attention, and their blood sugar levels should be closely monitored and strictly controlled due to the risk of rapid deterioration. It is also recommended that patients with underlying diseases take additional precautions to minimize the risk of infection. Doctors should closely monitor such patients for signs of disease progression.

### Retirement/Unemployment and COVID-19 Severity

Our results revealed that the retirement/unemployment was an independent risk factor for the severity of COVID-19, which is consistent with the findings of Liu et al. (Liu J. et al., 2020). This may be related to the older age of retirees and their underlying diseases, which are associated with poor prognosis (Peykari et al., 2015). Many unemployed individuals are women engaged in domestic work, and have a low education level, poor information access, weak awareness of epidemic prevention and control, and no stable economic source, which leads to delayed treatment of the disease. Therefore, the unemployed and retirees should be provided updated epidemic information and COVID-19 knowledge. This will allow them to detect symptoms in a timely manner, seek medical treatment as soon as possible, and prevent their illness from getting worse.

### Transmission Characteristics and Implications for Prevention of COVID-19

The source of infection was an independent factor affecting the severity of COVID-19 in our study. Here, 15.8 and 3.8% of patients with severe disease were from the Hubei Province and were from local residents respectively. Notably, in the early stage of the epidemic, the Hubei Province lacked medical resources and many patients could not receive timely treatment, leading to treatment delays (Zhang Z. Q. et al., 2020). We also found that the main sources of infection were family intimacy and close social contact (58.2%), followed by being in the epidemic area or having human contact history in Hubei Province (and 41.8%). The wave of outbreaks in China late 2019 and early 2020 began in the Hubei Province, especially in the city of Wuhan. Workers in the Hubei Province returned to their hometowns to celebrate the lunar new year, causing family infections. Because the Hubei residents were traveling or because of the COVID-19 outbreak, some non-Hubei residents who were in areas with shortages of local medical resources returned home for medical treatment, causing transmission during travel.

The results of this study are consistent with those of other studies showing that the main routes of transmission were close contact *via* respiratory droplets and close contact. However, contact or aerosol transmission could also be caused by exposure to virus-contaminated objects and environments under certain conditions (Kutti-Sridharan et al., 2020; Setti et al., 2020; Zhang R. et al., 2020).

When there is a COVID-19 epidemic in the community, the main measures to prevent the spread are to track and isolate close contacts of every COVID-19 patient and to diagnose or exclude COVID-19 infection through nucleic acid testing. Emphasis is placed on masks, personal hand hygiene, ventilation in the home and maintaining social distance as much as possible (Cowling et al., 2020).

### Smoking and COVID-19

The smoking, drinking, and BQ chewing rates in our study were 15.4, 26.4, and 7.1%, respectively. The chi-square test showed no statistical significance with the classification of COVID-19. The smoking rate of COVID-19 patients was lower than that the general population. No relationship was found between severity of COVID-19 and smoking, drinking alcohol, and BQ chewing in our study. Two studies (Guan et al., 2020b; Zhang J. J. et al., 2020) in China on COVID-19 and smoking showed that only 1 and 12.6% of patients were smokers, respectively, both lower than the smoking rate in our study (15.4%). Additionally, all of these rates were lower than the proportion of male smokers in China (>50%) (Stone et al., 2016; Qi et al., 2020). Therefore, it is not safe to speculate that whether smoking causes susceptibility to severe COVID-19. It should be also emphasize that due to the small sample size of our study, this conclusions need to be further confirmed by more sample size and multi-center studies.

The idea that smoking and alcohol consumption prevent COVID-19 has appeared in the media during the COVID-19 pandemic. One study reported (Luk et al., 2020) that 19.0% of respondents said they had seen claims in the media that smoking and alcohol consumption prevents COVID-19. There are even studies claiming that nicotine has a therapeutic effect on COVID-19. Nicotine is thought to have an anti-inflammatory effect, thereby regulating the body’s immune response and inhibiting the release of pro-inflammatory cytokines rather than anti-inflammatory cytokines such as IL-10 (Wang et al., 2003).

However, the World Health Organization (World Health Organization [WHO], 2020) states that smokers have a higher risk of developing severe COVID-19 and dying from COVID-19 and that there is not enough information to confirm any link between tobacco, nicotine, or alcohol consumption for the prevention or treatment of COVID-19. The WHO emphasizes that smokers’ hand-to-mouth behavior and smoke-induced lung diseases may increase their susceptibility to COVID-19 (World Health Organization [WHO], 2020).

Most studies have shown that smoking is related to the severity of COVID-19. Some (de Groot et al., 2019) have shown that smoking causes inflammation of the respiratory mucosa and lungs, and the chronic smoke stimulation of the respiratory tract causes COPD. These authors confirmed that smokers, including e-cigarette smokers, had higher levels of serum ACE2 expression (Brake et al., 2020) and increased susceptibility to COVID-19 caused by the upregulation of ACE2 (Li et al., 2020).

Every smoker should be encouraged to quit, and offered advice, support, and medication to help them quit. Times of crisis often provide the motivation to quit. We should make greater efforts to correct the erroneous view that smoking and alcohol consumption does not prevent COVID-19 from correcting erroneous views. Studies of smoking and COVID-19 require large samples or case-control studies to be able to identify confounding factors.

### Alcohol Consumption and COVID-19 Severity

In our study, all the drinkers except 4 frequent drinkers were social drinkers. Social alcohol consumption increases the possibility of gathering and spreading infectious diseases. We are cautious about alcohol consumption during the COVID-19 epidemic. Studies at the start of the COVID-19 outbreak showed that alcohol consumption could prevent COVID-19 from becoming an epidemic (Chick, 2020). Unfortunately, 180 Iranians drank methanol contaminated alcohol and died because they believed drinking could prevent COVID-19 (Delirrad and Mohammadi, 2020). Additionally, during the COVID-19 outbreak, people tend to drink because of increased stress caused by isolation and temporary absence from work. Close conversations while drinking, gathering in crowds, and even physical contact increase the spread of infectious diseases. Based on the prevailing evidence (Mungmungpuntipantip and Wiwanitkit, 2020), the World Health Organization (World Health Organization Regional Office for Europe, 2020) suggests that during the COVID-19 outbreak, only one alcoholic drink should be consumed per day.

### Betel Quid Chewing and COVID-19 Severity

At least 10% of the world’s population regularly chews BQ, which is the fourth most widely used addictive substance in the world (Sullivan and Hagen, 2002; Secretan et al., 2009). BQ chewing is popular among East Asians and their residents who migrate to other countries (Gupta and Warnakulasuriya, 2002; Changrani and Gany, 2005; Hashim et al., 2019). The Hunan Province in South China has the highest production and popular consumption of BQ, the rate of chewing BQ up to 38.4% (Xiao et al., 2011). Kaur and Rinkoo (2020) also noted that smokeless tobacco, especially betel quid, is popular in China and other parts of Southeast Asia. BQ affects cardiovascular, metabolic, respiratory, and reproductive health (Garg et al., 2014). Studies have also linked smokeless tobacco use, such as BQ chewing, to an increased risk of respiratory disease (Mehrtash et al., 2017). Alkaloids contained in areca nuts are nitrified to form N-nitrosamines, which may have toxic effects on human cells (No authors listed, 1985). In the Hunan Province China, 42.4% of BQ chewers also smoke (Zhang et al., 2008). Most people, who smoke, chew BQ, and drink alcohol, do not realize that their lifestyle is putting their health at serious risk. Chewing BQ leads to oral mucous membrane fibrosis, hyperkeratosis and ulcers (Chen et al., 2006; Song et al., 2015; Nithya et al., 2019). Research by Reichart et al. (2002) has shown that BQ users have a higher rate of oral lesions, and a high proportion of betel chewer’s have betel chewer’s mucosa (85.4%). Such oral lesions, cause atrophy of the epithelium and decreased blood vessels (Sharma et al., 2019). This not only directly leads to reduced local oral defense, but also damages a variety of immune populations including CD3 cells, CD4 cells, B lymphocytes, and macrophages, allowing for the invasion of pathogens (Pillai et al., 1990). Previous studies (Chang et al., 2005; Reichart et al., 2005; Singh et al., 2012) have indicated that consumption of BQ is strongly associated with HPV, Candida infection, tuberculosis, dengue fever, malaria, typhoid fever, HIV/AIDS, and other infectious diseases. The association between BQ consumption and pathogens such as SARS-CoV-2, including disease severity, is of concern.

The World Health Organization Framework Convention on Tobacco Control (Yach, 2003) provides evidence-based policies to reduce tobacco use, but a global policy to control BQ use is lacking. There is no smokeless tobacco legislation at the national level, and some developed countries may have overlooked the health risks of BQ chewing by immigrants (Lechner et al., 2019). During the COVID-19 pandemic, BQ chewing has increased opportunities for close interpersonal communication and aggregation. Hand-passing BQ increases the chances of virus contamination and transmission to others. Additionally, the increase in saliva caused by chewing may lead to spitting and saliva transmission. Research into the potential link between smokeless tobacco and COVID-19 must be prioritized for evidence-based policy development.

Smoking, drinking and BQ chewing are population-based disease risk factors, and these behavior increases the population agglomeration. The possibility of contact the pandemic may strengthen the behavior; therefore, the government should formulate policies, warn of the dangers of smoking, drinking and BQ chewing, and help affected individuals correct their behavior.

### Limitation

In this single-center retrospective cross-sectional study, COVID-19 patients who smoked, consumed alcohol, and chewed BQ accounted for a relatively small proportion of the total sample, and the relationship of these behaviors with the severity of COVID-19 may be affected by other confounding factors. Although all COVID-19 patients in the hospital were included in this study, the study sample is only taken from one hospital center and is relatively small which may indicate under representation of important factors, therefore the result**s** might not be generalizable to the wider population. How these factors interact with COVID-19 requires large population and case-control studies for further exploration in the future.

## Conclusion

This study indicates that there is an increased risk of severe COVID-19 among retired and unemployed individuals. Patients with diabetes and those from Wuhan, or the rest part of the Hubei province in the early stage or who traveled to other areas without timely treatment.

Retired/unemployed individuals and people with underlying diseases should be informed of methods of personal protection, and doctors should prevent these individuals from developing serious diseases. In addition, in clinical practice, it is important to pay attention to the source of infection and timely medical treatment.

## Data Availability Statement

The original contributions presented in the study are included in the article/supplementary material, further inquiries can be directed to the corresponding author/s.

## Ethics Statement

This study was approved by the Ethics Committee of Hunan Cancer Hospital (Approval Number: SBQLL-2020-094). Written informed consent to participate in this study was provided by the participants’ legal guardian/next of kin.

## Author Contributions

RZ and LC wrote this article. WW and YZ supervised the entire work and critically revised the manuscript. Data collection and statistics analysis by RZ, QZ, and YQ. All authors read and amended the final manuscript.

## Conflict of Interest

The authors declare that the research was conducted in the absence of any commercial or financial relationships that could be construed as a potential conflict of interest.
